# A Rapid LC-MS/MS Method for Simultaneous Determination of Ten Flavonoid Metabolites of Naringin in Rat Urine and Its Application to an Excretion Study

**DOI:** 10.3390/foods11030316

**Published:** 2022-01-24

**Authors:** Xuan Zeng, Yuying Zheng, Yan He, Wei Peng, Weiwei Su

**Affiliations:** Guangdong Engineering & Technology Research Center for Quality and Efficacy Reevaluation of Post-Market Traditional Chinese Medicine, Guangdong Provincial Key Laboratory of Plant Resources, School of Life Sciences, Sun Yat-sen University, Guangzhou 510275, China; zengx6@mail2.sysu.edu.cn (X.Z.); vicky_0224@126.com (Y.Z.); heyan53@mail2.sysu.edu.cn (Y.H.); pweiyu929@126.com (W.P.)

**Keywords:** naringin, metabolites, excretion, LC-MS/MS

## Abstract

Naringin is a dietary flavonoid glycoside with multiple bioactivities. It has been involved in numerous metabolism and excretion studies, and its metabolic properties are clear. However, information concerning the excretion profile of its original metabolites are still scarce, and few methods for simultaneous determination of multiple original metabolites of naringin in biological samples have been reported so far. In this study, a rapid and sensitive method for simultaneous determination of ten flavonoid metabolites of naringin in rat urine was developed with an UHPLC-Q-Trap-MS/MS system. One-step protein precipitation method with acetonitrile was used to extract analytes. A rapid chromatographic separation within 11 min was performed on an ACQUITY UPLC^®^ BEH C_18_ column (2.1 mm × 50 mm, 1.7 μm) using gradient elution with a mobile phase of water and methanol, both with 0.1% formic acid (*v*/*v*). MS/MS detection was conducted in negative ion mode and multiple reactions monitoring scanning mode. The analytical method was fully validated and successfully applied to monitor the excretion profiles of naringin in rat urine. Quantitative results revealed the visible individual difference and low urinary recovery of flavonoid metabolites in the excretion of naringin, which may be helpful for further study to understand the in vivo behavior and action mechanism of naringin.

## 1. Introduction

Naringin, also known as naringenin-7-*O*-neohesperidoside, is a dietary flavonoid glycoside widely distributed in citrus fruits [1]. It has been documented to exert multiple bioactivities of antioxidation, anti-inflammation, and anti-apoptosis [2]. A number of preclinical studies and clinical trials have further illustrated the benefits of naringin in relieving neurodegeneration [3], metabolic syndrome [4], cardiovascular disorders [5], and respiratory diseases [6]. In fact, naringin is increasingly employed as a typical phytopharmaceutical in the production of dietary supplements [7].

Profiling of metabolic and excretive properties is useful to understand the in vivo behavior and action mechanism of dietary phytochemicals [8]. Moreover, the coupling of liquid chromatography (LC) to tandem mass spectrometry (MS/MS) greatly facilitates metabolite identification and quantitation by combining the separating power of LC with the highly sensitive and selective analysis capability of MS/MS [9,10]. Thanks to its favorable bioactivities, naringin has been involved in numerous metabolism and excretion studies [11,12,13,14]. In previous studies, we have systemically investigated the absorption, tissue distribution, metabolism, and excretion of naringin in rats [15,16,17]. Naringin was found to be extensively hydrolyzed to naringenin, making its prototype almost undetectable in rat plasma and urine samples. Generated naringenin subsequently engaged in dehydrogenation, hydroxylation, methylation, glucuronidation, and sulfation, giving rise to a mass of metabolites. Glucuronides and sulfates of naringenin, apigenin, eriodictyol, and hesperetin were assigned as the primary metabolites of naringin in rat urine [16]. Although the metabolite profiles of naringin are clear, previous quantitative studies have mostly focused on free naringin and naringenin obtained after the co-incubation with glucuronidase and/or sulfatase, resulting in the loss of information concerning the excretion profile of original metabolites, especially conjugates such as glucuronides and sulfates. In fact, there are few reports on developing and validating methods for simultaneous determination of multiple original metabolites derived from naringin in biological samples so far. Given naringin is widely present in plant food, it is meaningful to develop a feasible method for monitoring the excretion profiles of multiple metabolites. In addition, this method would provide reference for studies concerning the excretion of other flavonoids.

**Figure 1:**
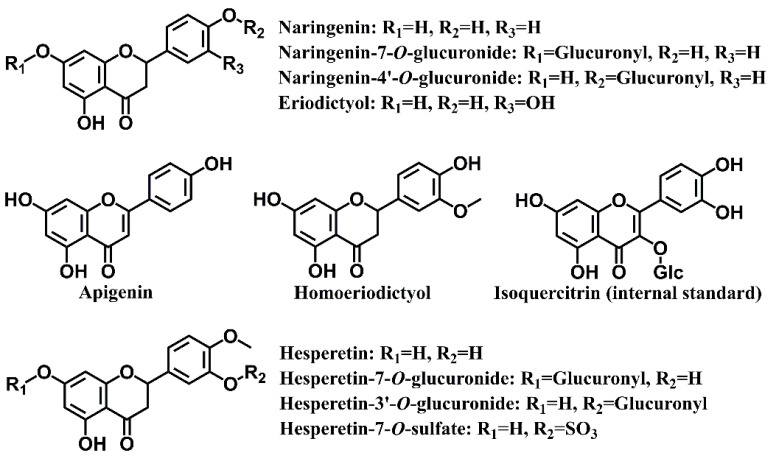
The chemical structures of target compounds determined in this work.

On the basis of previous results and the availability of reference substances, we selected ten compounds as the target metabolites of naringin in rat urine, including naringenin, naringenin-7-*O*-glucuronide, naringenin-4′-*O*-glucuronide, apigenin, eriodictyol, homoeriodictyol, hesperetin, hesperetin-7-*O*-glucuronide, hesperetin-3′-*O*-glucuronide, and hesperetin-7-*O*-sulfate (Figure 1). A rapid, simple, and sensitive method for simultaneous determination of these ten flavonoid metabolites was developed with ultra-high-performance liquid chromatograph coupled with hybrid triple quadrupole linear ion trap mass spectrometer (UHPLC-Q-Trap-MS/MS). The methodology was fully validated with selectivity, carry-over effect, calibration curve, lower limits of quantification (LLOQ), precision, accuracy, matrix effect, dilution integrity, and stability. The validated method was then successfully applied to investigate the excretion profiles of naringin in rat urine.

## 2. Materials and Methods

### 2.1. Chemicals and Reagents

The chemical reference substances of naringenin and hesperetin were obtained from Sigma-Aldrich (St. Louis, MO, USA). Naringenin-7-*O*-glucuronide and naringenin-4′-*O*-glucuronide was acquired from Cayman Chemical Company (Ann Arbor, MI, USA). Hesperetin-7-*O*-glucuronide, hesperetin-3′-*O*-glucuronide, and hesperetin-7-*O*-sulfate were purchased from Toronto Research Chemicals Inc. (Toronto, ON, Canada). Apigenin was supplied by Shanghai Macklin Biochemical Co., Ltd. (Shanghai, China). Eriodictyol was obtained from Dalian Meilunbio Biochemical Co., Ltd. (Dalian, China). Homoeriodictyol was purchased from Extrasynthese Chemical Company (Genay, France). The internal standard (IS) isoquercitrin was obtained from National Institute for the Control of Pharmaceutical and Biological Products (Beijing, China).

Methanol of LC-MS grade was acquired from Fisher Scientific Inc (Fair Lawn, NJ, USA), and acetonitrile of HPLC grade was purchased from Honeywell B&J Chemicals Inc (Morris Plains, NJ, USA). Water was purified by the Milli-Q system (Millipore Corporation; Billerica, MA, USA). Naringin powder for intragastric administration in animals was extracted from *Citrus grandis* ‘Tomentosa’, whose purity was 98.8% determined by HPLC [16].

### 2.2. Preparation of Calibration Standard and Quality Control (QC) Samples

The reference substances of naringenin, naringenin-7-*O*-glucuronide, naringenin-4′-*O*-glucuronide, hesperetin, hesperetin-7-*O*-glucuronide, hesperetin-3′-*O*-glucuronide, hesperetin-7-*O*-sulfate, eriodictyol, homoeriodictyol, and the internal standard (IS) isoquercitrin were individually weighed and then dissolved in 50% methanol, to obtain the stock solutions at concentration of 1 mg/mL. The stock solution of apigenin was prepared at concentration of 0.4 mg/mL due to the low solubility. To afford series working solutions of calibration curves and quality control (QC), the stock solutions of the mentioned ten compounds were appropriately mixed and then gradually diluted with 50% methanol. The IS stock solution was diluted to a concentration of 1 μg/mL with acetonitrile, to be used as the working solution for protein precipitation. All working solutions were prepared before use.

The calibration standard samples were prepared by spiking 5 μL of corresponding working solution to 95 μL of blank urine and then vortexed for 3 min, to obtain calibration standard samples of 0.5, 1, 2.5, 7.5, 25, 75, 200, and 250 ng/mL for eriodictyol and hesperetin; 1, 2, 5, 15, 50, 150, 400, and 500 ng/mL for apigenin, homoeriodictyol, and hesperetin-7-*O*-sulfate; 2, 4, 10, 30, 100, 300, 800, and 1000 ng/mL for naringenin; 4, 8, 20, 60, 200, 600, 1600, and 2000 ng/mL for hesperetin-7-*O*-glucuronide and hesperetin-3′-*O*-glucuronide; as well as 20, 40, 100, 300, 1000, 3000, 8000, and 10,000 ng/mL for naringenin-7-*O*-glucuronide and naringenin-4′-*O*-glucuronide, respectively. In the same manner, the low-concentration QC (LQC), middle-concentration QC (MQC), high-concentration QC (HQC) samples were prepared at 1.5, 15, and 187.5 ng/mL for eriodictyol and hesperetin; 3, 30, and 375 ng/mL for apigenin, homoeriodictyol, and hesperetin-7-*O*-sulfate; 6, 60, and 750 ng/mL for naringenin; 12, 120, and 1500 ng/mL for hesperetin-7-*O*-glucuronide and hesperetin-3′-*O*-glucuronide; 60, 600, and 7500 ng/mL for naringenin-7-*O*-glucuronide and naringenin-4′-*O*-glucuronide, respectively.

### 2.3. Sample Preparation for LC-MS/MS Analysis

Rat urine samples were thawed at room temperature and then vortexed for 1 min before processing. An aliquot of 100 μL of urine sample was vortex-mixed with 200 μL acetonitrile (containing IS with a concentration of 1 μg/mL) and subsequently centrifuged at 13,000 rpm for 30 min, to obtain the supernatant for LC-MS/MS analysis. Calibration standard and QC samples prepared above were treated in the same way.

### 2.4. LC-MS/MS Conditions

Sample determination was conducted with an ultra-high-performance liquid chromatograph (UHPLC; Shimadzu, Kyoto, Japan) coupled with a hybrid triple quadrupole linear ion trap mass spectrometer (QTRAP 6500^+^; Sciex, Framingham, MA, USA). The UHPLC system consisted of a communications bus module (CBM-20A), a degassing unit (DGU-20A), a binary pump (LC-30AD), an autosampler (SIL-30AC), and a column oven (CTO-20A), while the mass spectrometer was equipped with an electrospray ionization (ESI) source.

Chromatographic separation was carried out using on an ACQUITY UPLC^®^ BEH C18 column (2.1 mm × 50 mm, 1.7 μm; Waters, Milford, CT, USA) with the column temperature maintained at 40 °C. Water (A) and methanol (B), both containing 0.1% formic acid (*v*/*v*), were employed as the mobile phase, and eluted in accordance with following linear gradient elution program: 5% to 35% B at 0–7 min, 35–100% B at 7–11 min, and a 4 min post-run time to equilibrate the column. The flow rate was set at 0.4 mL/min. The injection volume was 10 μL, and the temperature of the autosampler was 15 °C. UHPLC effluent was directly injected into ESI source without splitting.

MS/MS detection was performed in negative ion mode and multiple reactions monitoring (MRM) scanning mode. Nitrogen was used as source gas, curtain gas, and collision gas, which was supplied continuously by a nitrogen generator (Genius NM32LA, Peak Scientific, USA). To obtain the optimal intensity, the common ion source parameters were set as follows: Curtain Gas (CUR) 35 psi, Collision Gas (CAD) medium, Ion Spray Voltage (ISV) −4500 eV, Temperature (TEM) 550 °C, Ion Source Gas 1 (GS1) 55 psi, and Ion Source Gas 2 (GS2) 55 psi. The MS/MS transitions and other optimized parameters, including Declustering Potential (DP) and Collision Energy (CE), were listed in Table 1. Some glucuronidated isomers were detected under the same parameters.

### 2.5. Method Validation

The present method was developed and validated in accordance with the Guidelines for Validation of Quantitative Analytical Method of Biological Samples documented in the Pharmacopoeia of the People’s Republic of China (ChP) 2020 edition [18]. The validation mainly focused on selectivity, carry-over effect, calibration curve, LLOQ, precision, accuracy, matrix effect, dilution integrity, and stability.

#### 2.5.1. Selectivity

The selectivity of this method was assessed by comparing the MRM chromatograms of blank rat urine, blank rat urine spiking target compounds and IS, and rat urine sample collected after the oral administration of naringin, so as to confirm that there was neither endogenous matrix interference nor mutual interference between target compounds.

#### 2.5.2. Carry-Over Effect

The carry-over effect was evaluated by injecting the blank urine sample after assaying the upper limit of quantification (ULOQ) sample. In principle, the residual response of each analyte in blank urine sample should not exceed 20% of the LLOQ sample, as well as no more than 5% for IS.

#### 2.5.3. Calibration Curve and LLOQ

A batch of calibration standard samples was composed of a blank urine sample (blank urine processed without analytes and IS), a zero-concentration urine sample (blank urine processed without analytes but with IS), and eight concentration levels of samples (blank urine processed with the working solutions of calibration curves and IS) covering corresponding linear range. The calibration curves were constructed by plotting the peak area ratio of analyte relative to IS (y) against the analyte concentrations (x) with a weighted (1/x) least square linear regression. LLOQ was the lowest concentration of the calibration curve. The deviation of measured concentration from the nominal value for LLOQ samples should be within ±20%, while that for other calibration standard samples should be within ±15%. The deviation can be calculated by the following equation: Deviation = 100% × (Measured concentration − Nominal value)/Nominal value.

#### 2.5.4. Precision and Accuracy

Precision and accuracy were evaluated by analyzing six replicates of QC samples at four concentration levels (LLOQ, LQC, MQC, HQC) in the same batch (intra-batch) and between three different batches completed in no less than two days (inter-batch). The concentrations of these QC samples were calculated with the calibration curve constructed in the same batch. Intra-batch and inter-batch precision were expressed in terms of RSD (%), while the accuracy was calculated by comparing acquired concentrations with the nominal values and presented as relative error (RE, %). The RSD of LLOQ samples should be within 20%, and that for LQC, MQC, and HQC samples should be not exceed 15%. The acceptance criterion of RE for LLOQ samples was ±20%, while that for other QC samples was ±15%.

#### 2.5.5. Matrix Effect

The matrix effect was assessed by determining LQC and HQC samples (n = 3) prepared with blank rat urine from six different sources (three replicate samples per source). The matrix factor (MF) of target analyte and IS were obtained by comparing the peak area of post-extraction spiked samples with that acquired in neat solution (i.e., MF = Peak area _post-extraction spiked sample_/Peak area _neat solution_), while the IS-normalized matrix factor (IS-MF) was calculated as the ratio of analyte’s MF against IS’s MF (i.e., IS-MF = MF_analyte_/MF_IS_). The acceptance criterion for the RSD (%) of IS-MF was within 15%.

#### 2.5.6. Dilution Integrity

The dilution integrity tests were performed with the aim to verify the reliability of the dilution process for samples at higher concentrations above ULOQ, which may be encountered during sample analysis. Three replicates of samples with concentrations multiple times (5 times, 10 times) higher than HQC were prepared by spiking high concentration analyte to blank urine, and then diluted to the HQC concentration level. The accuracy of the diluted sample was acceptable within ±15% of the nominal value.

#### 2.5.7. Stability

The stability of the target analytes in rat urine were evaluated by analyzing three replicates of QC samples at two concentrations (LQC and HQC). Long-term stability was assayed after being stored at −70 °C for one month and three months. Freeze–thaw stability was determined after being subjected to two freeze (at −70 °C) and thaw (at room temperature) cycles. The placement stability of treated sample in autosampler was examined after being maintained under autosampler conditions (15 °C) for 24 h. For all QC samples in the stability test, the concentrations were calculated with freshly prepared calibration curve. The RSD% and RE% of calculated concentration values should be within the range of ±15%.

### 2.6. Excretion Study

The excretion study was approved by the Institutional Animal Care and Use Committee (IACUC) in Sun Yat-Sen University and conducted in the light of relevant guidelines for the care and use of laboratory animals. Five male Sprague-Dawley rats (weighing 300–400 g) were purchased from Guangdong Medical Laboratory Animal Center (Guangzhou, China). Animals were housed in controlled environment with temperature at 20–23 °C, relative humidity at 55 ± 5%, light–dark cycle of 12/12 h, as well as free access to standard feed and water. After fasting for 12 h, rats were administrated intragastrically with naringin at the dosage of 42 mg/kg, and then housed individually in metabolic cages (Y-3102, Yuyan Instruments Co. Ltd.; Shanghai, China) with water supplied *ad libitum* but no food. Urine samples were collected over six periods of 0–4, 4–8, 8–12, 12–24, 24–36, and 36–48 h post dose, and their volume were measured with a graduated cylinder. Obtained urine samples were reserved at −70 °C until analysis.

### 2.7. Data Analysis

Raw mass spectrometer data were acquired with SCIEX Analyst software (Version 1.7 with HotFix 3) and then processed with SCIEX OS software (Version 1.4.1.20719). Figures were plotted with GraphPad Prism software (Version 7.00) and Microsoft Excel software (Version 2016).

## 3. Results and Discussion

### 3.1. Method Development

In this work, both mass spectrometry parameters and chromatographic conditions were optimized in method development. To obtain sensitive and stable responses, working solutions of target analytes (100 ng/mL) prepared in methanol were infused directly into the ESI source in positive and negative ionization modes, respectively. The negative ionization mode was selected on account of the higher and more stable signal strengths. Subsequently, the MS/MS ion transitions were screened in MRM scanning mode to enhance the selectivity of detection. The most responsive ion transitions were observed at *m*/*z* 270.9 to 150.9 for naringenin, *m*/*z* 447.0 to 271.1 for naringenin-7-*O*-glucuronide and naringenin-4′-*O*-glucuronide, *m*/*z* 269.0 to 151.0 for apigenin, *m*/*z* 286.9 to 135.0 for eriodictyol, *m*/*z* 301.0 to 150.9 for homoeriodictyol, *m*/*z* 301.0 to 163.9 for hesperetin, *m*/*z* 477.1 to 301.1 for hesperetin-7-*O*-glucuronide and hesperetin-3′-*O*-glucuronide, *m*/*z* 381.0 to 301.1 for hesperetin-7-*O*-sulfate, and *m*/*z* 463.1 to 299.9 for isoquercitrin (IS), respectively. Additionally, the DP and CE parameters of target analytes were also optimized to acquire the richest relative abundance of the precursor and product ions.

As to the chromatographic conditions, different columns (Waters ACQUITY UPLC^®^ BEH C_18_ column, Phenomenex Kinetex C_18_ column, and Agilent Poroshell 120 EC-C_18_ column), composition of mobile phases (acetonitrile–water, methanol–water, with or without formic acid), column temperatures (20, 30, and 40 °C), flow rates (0.2, 0.3, and 0.4 mL/min), as well as elution programs (gradient time, gradient shape, and initial composition of the mobile phase) were optimized to obtain chromatograms with good shape. After continuous optimization, a 11 min gradient elution program was finally performed on an ACQUITY UPLC^®^ BEH C_18_ column (2.1 mm × 50 mm, 1.7 μm) with water–methanol (both containing 0.1% formic acid) as the mobile phase, column temperature at 40 °C, and flow rate at 0.4 mL/min.

### 3.2. Method Validation

#### 3.2.1. Selectivity

**Figure 2:**
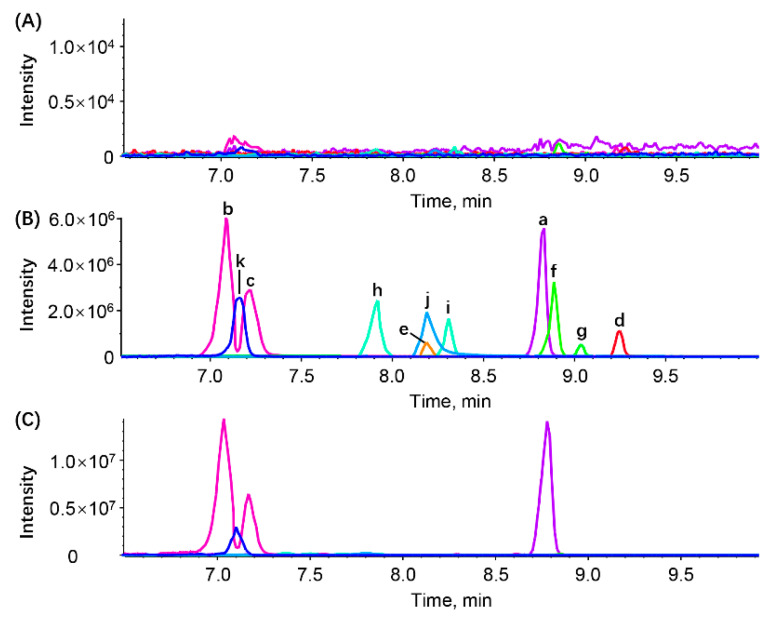
Typical MRM chromatogram of naringenin (a, RT = 8.82 min, purple), naringenin-7-*O*-glucuronide (b, RT = 7.09 min, pink), naringenin-4′-*O*-glucuronide (c, RT = 7.23 min, pink), apigenin (d, RT = 9.25 min, red), eriodictyol (e, RT = 8.19 min, orange), homoeriodictyol (f, RT = 8.89 min, pale green), hesperetin (g, RT = 9.03 min, green), hesperetin-7-*O*-glucuronide (h, RT = 7.91 min, aquamarine blue), hesperetin-3′-*O*-glucuronide (i, RT = 8.31 min, aquamarine blue), and hesperetin-7-*O*-sulfate (j, RT = 8.20 min, blue), and IS (k, RT = 7.15 min, indigo) in blank urine (**A**); simulated urine (blank urine spiked with IS and analytes at HQC concentration level) (**B**); and actual urine sample (collected from rat 3 at 8–12 h after the oral administration of naringin) (**C**).

The typical MRM chromatograms of target analytes and IS in blank urine, simulated urine (blank urine spiked with analytes and IS), and actual urine sample (collected after the oral administration of naringin) were shown in Figure 2. The retention time (RT) of naringenin, naringenin-7-*O*-glucuronide, naringenin-4′-*O*-glucuronide, apigenin, eriodictyol, homoeriodictyol, hesperetin, hesperetin-7-*O*-glucuronide, hesperetin-3′-*O*-glucuronide, hesperetin-7-*O*-sulfate, and IS were 8.82, 7.09, 7.23, 9.25, 8.19, 8.89, 9.03, 7.91, 8.31, 8.20, and 7.15 min, respectively. There observed no interference peaks within the RT range of the analyte and IS, indicating the developed method was highly specific for the detection of target analytes in the rat urine sample.

#### 3.2.2. Carry-Over Effect

The residual responses of the ten target analytes in blank urine detected after ULOQ sample were all within 20% of that in LLOQ, while that for IS was less than 5%. These results suggested that the carry-over effect of this method could be negligible in sample determination.

#### 3.2.3. Calibration Curve and LLOQ

The deviation of all calibration standard samples met the acceptance criteria, and all calibration curves of target analytes over the corresponding concentration range had correction coefficients greater than 0.99, showing good linearity (Table 2). The LLOQ of naringenin, naringenin-7-*O*-glucuronide, naringenin-4′-*O*-glucuronide, apigenin, eriodictyol, homoeriodictyol, hesperetin, hesperetin-7-*O*-glucuronide, hesperetin-3′-*O*-glucuronide, and hesperetin-7-*O*-sulfate were 2.140, 20.00, 20.00, 0.9430, 0.4464, 0.9900, 0.5510, 4.200, 3.600, and 0.9700 ng/mL, respectively. The intra-batch and inter-batch assay of LLOQ samples achieved acceptable precision (RSD) of <9.9%, and accuracy (RE) varied from −1.1% to 10.7% for target analytes.

#### 3.2.4. Precision and Accuracy

The precision and accuracy data for target analytes obtained from QC samples at four different concentration levels (LLOQ, LQC, MQC, HQC) were summarized in Table 3. The intra-batch and inter-batch precision (RSD) were <9.9% with accuracy (RE) in the range of −10.2% to 10.7% for all analytes. Obtained assay values were within the acceptable criteria, which revealed that the present method was stable, reliable, and reproducible for simultaneous determination of ten analytes in rat urine.

#### 3.2.5. Matrix Effect

The IS-normalized matrix factor (IS-MF) of target analytes tested at LQC and HQC concentration levels is shown in Table 3. Mean values of IS-MF for ten analytes varied from 0.93 to 1.14, with the precision of <7.5%. These data indicated that there were no significant matrix effects for any of the target analytes under current experimental conditions.

#### 3.2.6. Dilution Integrity

The dilution integrity tests were evaluated by diluting three replicates of the high concentration sample (5 or 10 times the concentration of HQC), and analyzing them at the concentration level of HQC. As shown in Table 4, the precision of ten target analytes diluted 5 times and 10 times was less than 7.6%, and their accuracy expressed as RE was in the range of −9.9% to 5.9%. These results suggested that the sample dilution process did not affect the reliability of quantitative results.

#### 3.2.7. Stability

The results of long-term stability, freeze–thaw stability, and placement stability in the autosampler were summarized in Table 4. At LQC and HQC concentration levels, the deviations between measured values and nominal values were all within the range from −9.3% to 10.7%, with the precision of <12.0%, revealing that all target analytes were stable in rat urine under mentioned storage and processing conditions.

### 3.3. Excretion Study

**Figure 3:**
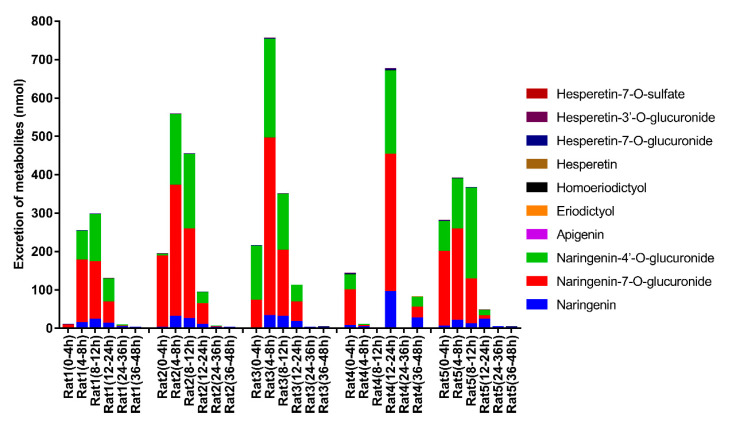
The urinary excretion profiles of ten flavonoid metabolites derived from naringin in rats.

The validated LC-MS/MS method was successfully applied to an excretion study of ten flavonoid metabolites in rat urine after the oral administration of naringin at a dose of 42 mg/kg. Measured concentrations of target analytes in rat urine are listed in Table S1 in the Supplementary Material, while the body weight and urine volume are shown in Table S2. These metabolites were not detected in the urine samples collected before the administration. The excretion profiles of these ten metabolites over six time periods are illustrated in Figure 3. Obviously, naringenin-7-*O*-glucuronide, naringenin-4′-*O*-glucuronide, and naringenin were the primary metabolites of naringin in rat urine. As shown in Figure 2, most metabolites were excreted in urine within 24 h post dose. The excretion profile of rat 4 was quite different from that of other rats, mainly due to the difference in urine volume. As shown in Table S2, the urine volume of rat 4 rat at 8–12 h and 24–36 h was zero, and that at 4–8 h was only 0.8 mL, which was far less than that of the other rats. Considering the corresponding body weight and urine volume, the overall urinary excretion of the ten flavonoid metabolites in the five rats was 691.6, 1296, 1428, 903.8, and 1083 nmol, which corresponded to 2.68%, 5.00%, 6.14%, 3.14%, and 4.42% of naringin intake, respectively. These results reflect the visible individual difference and low urinary recovery of flavonoid metabolites in the excretion of naringin.

Naringin is a flavanone-*O*-glycoside with the disaccharide neohesperidose bound to the C-7 position. Once entering the gut, naringin is subject to the hydrolysis mediated by lactase-phlorizin hydrolase and gut microbiota, losing the neohesperidose moiety and yielding the aglycone naringenin [19]. Naringenin is subsequently absorbed into the circulatory system and engaged in extensive phase I and phase II metabolism, giving rise to several other aglycones (apigenin, eriodictyol, homoeriodictyol, and hesperetin) and corresponding conjugates (glucuronides, sulfates) [16]. The enzymes involved in the above-mentioned metabolic process, such as lactase-phlorizin hydrolase, UDP-glucuronosyltransferases, and sulfotransferase, have been documented to have genetic polymorphisms [20,21,22]. The polymorphism of enzyme expression and activity undoubtedly affects the metabolism and excretion of naringin in vivo [23].

The gut microbiota is another important variable affecting the metabolism of naringin [24]. It is the prominent inhabitant of the gastrointestinal tract, and is influenced by diets, antibiotics, and other environmental factors [25]. The gut microbiota is deeply involved in the degradation of exogenous compounds in the digestive tract by secreting a mass of metabolic enzymes [26]. Take naringin as an example, the gut microbiota plays a pivotal role in the hydrolysis and subsequent rupture of the flavanone skeleton [27]. In a reported work, we detected a total of 46 microbial-derived phenolic catabolites in rat urine samples collected after the oral administration of naringin, including phenylpropenoic acid, phenylpropionic acid, phenylacetic acid, benzoic acid, and corresponding conjugates [16]. In fact, mediated by a large number of gut microbes, unabsorbed naringin and naringenin were extensively degraded into phenolic catabolites, resulting in the low recovery of naringin in rat urine [28,29]. Liu et al. determined the concentrations of naringin and naringenin in the rat urine samples after incubation with glucuronidase and sulfatase, and found that the cumulative excretions of naringin and total naringenin amounted to 5% of the ingested dose [15], which is similar to the results obtained in this study (the mean urinary recovery of naringin was 4.27%). Given that phenolic catabolites are an important part of naringin excretion, we have tried to detect them together with flavonoid metabolites. However, we found that there existed thorny endogenous interferences in the determination of phenolic catabolites, which meant they could be detected in blank urine samples. Herein, the endogenous interferences made it difficult to determine flavonoid metabolites and phenolic catabolites simultaneously. Recently, we have been trying to address this issue with a stable isotope labeling strategy.

## 4. Conclusions

In this study, a simple, sensitive, and reproducible LC-MS/MS method was developed and validated for the simultaneous determination of ten flavonoid metabolites derived from naringin in rat urine. With this method, ten analytes in rat urine could be simultaneously quantified within a short chromatographic elution time (11 min). It was successfully applied to determine naringin and its metabolites in rat urine after the oral administration of naringin. The obtained results suggest that there exists a visible individual difference and low urinary recovery of flavonoid metabolites in the excretion of naringin. It would be helpful for further study to understand the in vivo behavior and action mechanism of naringin. In addition, this method provides reference for studies concerning the excretion of other flavonoids.

## Figures and Tables

**Table 1:** The optimized MS/MS transitions, Declustering Potential (DP), and Collision Energy (CE) parameters for target analytes.

No.	Analytes	Q1 Mass (Da)	Q3 Mass (Da)	DP (eV)	CE (eV)
1	Naringenin	270.9	150.9	−24	−44
2	Naringenin-7-*O*-glucuronide, Naringenin-4′-*O*-glucuronide	447.0	271.1	−33	−72
3	Apigenin	269.0	151.0	−33	−48
4	Eriodictyol	286.9	135.0	−38	−56
5	Homoeriodictyol	301.0	150.9	−26	−50
6	Hesperetin	301.0	163.9	−32	−134
7	Hesperetin-7-*O*-glucuronide, Hesperetin-3′-*O*-glucuronide	477.1	301.1	−31	−59
8	Hesperetin-7-*O*-sulfate	381.0	301.1	−28	−53
9	Isoquercitrin (IS)	463.1	299.9	−36	−32

**Table 2:** The correlation coefficients (r) and linear ranges of calibration curves.

No.	Analytes	r	Linear Ranges (ng/mL)
1	Naringenin	0.99072	2.140~1070
2	Naringenin-7-*O*-glucuronide	0.99350	20.00~10,000
3	Naringenin-4′-*O*-glucuronide	0.99006	20.00~10,000
4	Apigenin	0.99320	0.9430~471.5
5	Eriodictyol	0.99748	0.4464~223.2
6	Homoeriodictyol	0.99574	0.9900~495.0
7	Hesperetin	0.99004	0.5510~275.5
8	Hesperetin-7-*O*-glucuronide	0.99681	4.200~2100
9	Hesperetin-3′-*O*-glucuronide	0.99316	3.600~1800
10	Hesperetin-7-*O*-sulfate	0.99252	0.9700~485.0

**Table 3:** Intra-batch and inter-batch precision, accuracy, and IS-normalized matrix factor (IS-MF) of target analytes in rat urine.

Analytes	Conc. (ng/mL)	Intra-Batch (n = 6)		Inter-Batch (n = 6 × 3)		IS-MF (n = 3 × 6)	
		RSD%	RE%	RSD%	RE%	Mean	RSD%
Naringenin	2.140	3.9	5.7	8.5	−1.1	-	-
	6.420	8.1	−2.5	7.2	−4.5	1.04	3.7
	64.20	6.3	−8.0	5.7	−5.6	-	-
	802.5	2.8	−1.1	6.6	−3.6	0.96	2.7
Naringenin-7-*O*-glucuronide	20.00	6.8	10.7	8.9	3.8	-	-
	60.00	4.2	2.6	6.1	−0.1	1.05	4.5
	600.0	7.0	−2.5	7.3	1.5	-	-
	7500	5.9	0.7	6.4	−0.2	0.93	2.5
Naringenin-4′-*O*-glucuronide	20.00	8.9	−0.3	9.2	2.5	-	-
	60.00	5.0	−4.8	6.7	−0.8	1.05	3.8
	600.0	3.4	−6.1	5.5	−1.8	-	-
	7500	4.0	0.7	6.4	−0.7	0.95	3.9
Apigenin	0.9430	5.8	4.0	8.7	0.5	-	-
	2.829	7.9	−6.5	6.6	−6.0	1.05	4.4
	28.29	6.5	−10.2	4.8	−7.6	-	-
	353.6	3.4	−2.5	6.3	−3.3	0.98	3.5
Eriodictyol	0.4464	8.5	2.8	7.4	4.0	-	-
	1.339	5.5	−2.6	5.6	−3.2	1.12	2.2
	13.39	4.1	−5.8	6.1	−1.4	-	-
	167.4	6.5	−2.7	6.6	−0.9	0.98	1.7
Homoeriodictyol	0.9900	4.6	6.8	7.5	1.9	-	-
	2.970	7.2	4.6	7.7	−2.9	1.01	5.8
	29.70	5.1	−8.0	5.3	−5.9	-	-
	371.3	3.0	0.1	5.8	−2.4	0.98	3.9
Hesperetin	0.551	3.8	7.6	4.0	8.6	-	-
	1.653	6.2	−4.2	6.1	−2.8	1.09	4.7
	16.53	5.4	−6.3	6.3	−2.8	-	-
	206.6	2.6	0.2	5.5	−1.1	1.08	2.2
Hesperetin-7-*O*-glucuronide	4.200	6.3	4.3	7.7	3.3	-	-
	12.60	5.0	−4.9	6.4	0.4	1.00	7.4
	126.0	6.0	−9.1	6.8	−2.4	-	-
	1575	4.6	−1.1	5.2	1.7	0.95	3.2
Hesperetin-3′-*O*-glucuronide	3.600	3.2	7.5	7.5	1.5	-	-
	10.80	6.0	0.6	6.5	−0.2	1.06	4.0
	108.0	6.7	−5.5	7.4	−2.9	-	-
	1350	2.5	3.0	5.2	3.8	1.00	2.7
Hesperetin-7-*O*-sulfate	0.9700	9.8	2.3	8.3	2.2	-	-
	2.910	8.8	3.4	8.6	−0.3	1.14	2.1
	29.10	8.0	−4.2	7.5	−1.5	-	-
	363.8	6.4	5.6	5.2	3.9	0.93	5.1

**Table 4:** Stability and dilution integrity of target analytes in rat urine under different conditions (n = 3).

Analytes	Conc. (ng/mL)	Long Term (−70 °C, 1 Month)		Long Term (−70 °C, 3 Months)		Freeze–Thaw (1 Cycle)		Freeze–Thaw (2 Cycles)		Placement in Autosampler (15 °C, 24 h)		Dilution Integrity (5 Times)		Dilution Integrity (10 Times)	
		RSD%	RE%	RSD%	RE%	RSD%	RE%	RSD%	RE%	RSD%	RE%	RSD%	RE%	RSD%	RE%
Naringenin	6.420	5.0	2.3	4.0	−2.0	4.0	2.9	12.0	−1.9	4.0	8.2	-	-	-	-
	802.5	1.8	0.3	7.1	−1.1	7.1	−4.7	4.2	0.6	1.8	3.1	5.0	0.3	0.9	−0.2
Naringenin-7-*O*-glucuronide	60.00	5.2	6.9	6.4	4.3	4.9	−1.5	6.9	4.1	3.8	10.5	-	-	-	-
	7500	7.0	−5.5	6.7	−2.2	3.9	−4.4	6.7	−9.3	7.5	−4.6	2.8	−7.0	5.9	−9.9
Naringenin-4′-*O*-glucuronide	60.00	6.3	0.4	5.1	2.3	11.0	3.8	3.0	8.0	7.1	5.4	-	-	-	-
	7500	8.2	−3.7	6.8	1.4	6.5	−3.7	4.5	2.1	4.4	6.1	1.6	0.1	5.7	0.7
Apigenin	2.829	3.6	−1.1	6.7	1.4	9.4	−0.1	8.1	7.6	3.4	9.3	-	-	-	-
	353.6	1.8	0.6	6.5	1.2	6.3	−7.0	5.4	2.3	2.6	1.3	5.8	−1.1	4.3	−3.1
Eriodictyol	1.339	5.5	0.1	9.1	0.9	7.5	1.6	11.3	−3.9	5.9	−2.3	-	-	-	-
	167.4	3.4	−2.3	6.1	−1.8	7.1	−6.4	4.2	−3.2	6.4	−3.9	5.6	−0.7	5.6	−0.4
Homoeriodictyol	2.970	10.7	−3.7	7.7	4.1	3.8	10.7	9.8	5.8	9.4	2.3	-	-	-	-
	371.3	3.3	3.5	9.0	−1.8	8.0	−6.0	2.1	4.1	1.3	1.4	6.6	−0.7	5.0	1.1
Hesperetin	1.653	10.4	−1.1	6.9	−2.4	3.3	6.6	9.7	4.3	6.2	7.5	-	-	-	-
	206.6	4.3	1.1	5.4	0.4	7.8	−4.7	1.2	−1.0	5.6	−1.4	7.5	0.7	5.2	−0.9
Hesperetin-7-*O*-glucuronide	12.60	5.2	−6.0	7.9	−7.4	10.0	−1.3	3.6	−0.5	5.1	4.5	-	-	-	-
	1575	4.7	1.1	6.7	−2.4	7.3	−6.4	2.9	−1.6	4.0	2.3	2.9	0.0	4.4	−3.1
Hesperetin-3′-*O*-glucuronide	10.80	2.1	2.9	11.3	0.5	4.6	7.2	9.1	3.8	6.8	−3.9	-	-	-	-
	1350	1.8	4.0	8.2	0.0	7.2	−0.6	3.4	6.1	5.6	1.1	2.0	−1.5	2.7	5.6
Hesperetin-7-*O*-sulfate	2.910	2.7	3.8	10.4	2.4	10.2	2.0	8.2	4.7	8.6	0.8	-	-	-	-
	363.8	4.3	−2.5	7.3	−1.5	8.9	−4.2	1.6	8.3	8.8	−4.9	6.5	5.9	7.3	−0.1

## Data Availability

Data is contained within the article.

## Supplementary Materials

The following supporting information can be downloaded at: https://www.mdpi.com/article/10.3390/foods11030316/s1, Table S1: Measured concentrations (ng/mL) of target analytes in rat urine samples collected after the oral administration of naringin; Table S2: Body weights (g) and urine sample volume (mL) of rats.
