# Supplementary material for: A Rapid LC-MS/MS Method for Simultaneous Determination of Ten Flavonoid Metabolites of Naringin in Rat Urine and Its Application to an Excretion Study
Source: Foods. 2022 Jan 24;11(3):316. doi: 10.3390/foods11030316 (PMC8834102; doi:10.3390/foods11030316)
Supplement: Supplementary file 1 [file foods-11-00316-s001.zip › foods-1531271-supplementary.pdf]

Table S1. Measured concentrations (ng/mL) of target analytes in rat urine samples collected after the oral administration of naringin.

Samples		Naringenin	Naringenin-7-*O*-glucuronide	Naringenin-4′-*O*-glucuronide	Apigenin	Eriodictyol	Homoeriodictyol	Hesperetin	Hesperetin-7-*O*-glucuronide	Hesperetin-3′-*O*-glucuronide	Hesperetin-7-*O*-sulfate
Rat 1	0-4 h	< LLOQ	1284	< LLOQ	< LLOQ	0.6269	< LLOQ	< LLOQ	82.25	11.14	< LLOQ
	4-8 h	765.2	17086	7755	10.17	1.321	3.512	2.108	96.63	57.57	1.193
	8-12 h	1206	13420	10944	12.90	1.605	6.937	3.284	77.41	67.71	1.689
	12-24 h	432.6	3572	3811	4.637	1.277	3.745	1.576	35.44	24.77	1.169
	24-36 h	243.4	299.1	473.2	1.396	0.9778	3.152	0.9564	21.54	8.888	1.805
	36-48 h	44.68	< LLOQ	< LLOQ	< LLOQ	0.9180	1.254	1.062	< LLOQ	< LLOQ	< LLOQ
Rat 2	0-4 h	131.7	29596	634.9	6.253	1.021	1.501	< LLOQ	232.7	27.38	1.265
	4-8 h	2734	52822	28299	13.17	3.033	9.191	4.049	129.8	73.26	1.358
	8-12 h	1626	26043	21898	8.266	1.874	9.096	2.445	69.58	50.43	2.427
	12-24 h	183	2041	1125	< LLOQ	< LLOQ	1.850	< LLOQ	7.410	5.126	< LLOQ
	24-36 h	70.62	128.2	93.39	< LLOQ	0.9825	1.105	< LLOQ	8.927	< LLOQ	1.604
	36-48 h	15.74	< LLOQ	< LLOQ	< LLOQ	0.8825	< LLOQ	< LLOQ	< LLOQ	< LLOQ	< LLOQ
Rat 3	0-4 h	15.48	10519	21176	1.015	0.5321	< LLOQ	< LLOQ	120.1	24.13	1.298
	4-8 h	2094	50541	28048	19.37	4.209	10.24	3.359	168.7	108.8	2.998
	8-12 h	2051	20383	17137	18.19	4.221	15.94	3.115	83.10	68.29	2.491
	12-24 h	242.1	1305	1104	2.160	0.8219	3.312	< LLOQ	7.611	6.621	< LLOQ
	24-36 h	24.56	< LLOQ	< LLOQ	< LLOQ	0.9085	1.169	< LLOQ	5.254	4.079	1.248
	36-48 h	77.98	< LLOQ	< LLOQ	1.243	1.416	1.734	1.316	< LLOQ	< LLOQ	1.601
Rat 4	0-4 h	250.1	6899	2964	5.610	2.271	5.966	1.850	191.4	46.17	< LLOQ
	4-8 h	740.8	1779	1553	7.252	2.480	15.49	5.018	47.09	16.05	< LLOQ
	8-12 h	No sample									
	12-24 h	2860	18013	10901	20.07	6.182	20.77	8.269	192.1	57.30	1.260
	24-36 h	No sample									

	36-48 h	1236	2213	2058	8.237	5.657	15.92	5.626	48.96	23.49	2.073
Rat 5	0-4 h	208.5	17046	6899	3.390	3.489	4.132	0.9285	160.3	87.42	1.266
	4-8 h	822.9	16648	9108	6.791	1.638	5.409	2.462	57.24	61.06	1.134
	8-12 h	656.4	13865	27908	6.416	1.417	5.436	2.056	65.61	42.54	< LLOQ
	12-24 h	376.7	238.3	406.8	2.659	1.306	7.105	1.592	7.245	4.618	< LLOQ
	24-36 h	92.65	< LLOQ	< LLOQ	1.965	1.342	6.087	0.7865	5.876	< LLOQ	< LLOQ
	36-48 h	37.74	< LLOQ	< LLOQ	3.692	1.959	4.863	0.7631	< LLOQ	< LLOQ	< LLOQ

Table S2. Body weights (g) and urine sample volume (mL) of rats.

Animals		Rat 1	Rat 2	Rat 3	Rat 4	Rat 5
Body weiths (g)		356.5	358.4	321.5	398.1	338.1
Urine sample volume (mL)	0-4 h	2.5	2.8	3.0	6.0	5.1
	4-8 h	4.3	2.9	4.1	0.8	6.4
	8-12 h	5.0	4.0	3.8	0.0	3.8
	12-24 h	7.0	11.8	17.5	8.9	15.8
	24-36 h	2.6	4.2	6.0	0.0	6.6
	36-48 h	6.6	10.2	5.0	5.6	8.6
